# Treatment of Periprosthetic Infections: An Economic Analysis

**DOI:** 10.1155/2013/821650

**Published:** 2013-05-28

**Authors:** Daniel Hernández-Vaquero, Mariano Fernández-Fairen, Ana Torres, Ann M. Menzie, José Manuel Fernández-Carreira, Antonio Murcia-Mazon, Enrique Guerado, Luis Merzthal

**Affiliations:** ^1^Hospital St Agustín and School of Medicine, 33400 Oviedo, Spain; ^2^Orthopaedic Surgery and Traumatology Institute, 08009 Barcelona, Spain; ^3^Hospital of Cartagena, 30203 Murcia, Spain; ^4^Hospital of Cabueñes and School of Medicine, 33394 Oviedo, Spain; ^5^Hospital Costa del Sol, 29603 Marbella, Spain; ^6^Hospital Sabogal, Colina 1081, Bellavista-Callao, Peru

## Abstract

This review summarizes the existing economic literature, assesses the value of current data, and presents procedures that are the less costly and more effective options for the treatment of periprosthetic infections of knee and hip. Optimizing antibiotic use in the prevention and treatment of periprosthetic infection, combined with systemic and behavioral changes in the operating room, the detection and treatment of high-risk patient groups, as well as the rational management of the existing infection by using the different procedures according to each particular case, could allow for improved outcomes and lead to the highest quality of life for patients and the lowest economic impact. Nevertheless, the costeffectiveness of different interventions to treat periprosthetic infections remains unclear.

## 1. Introduction

Infection is the cause of 14.8% of Total Hip Arthroplasty (THA) revisions [1] and the most common cause of Total Knee Arthroplasty (TKA) revisions (25.2%) [2]. At least one rehospitalization due to deep infection during the first year after primary THA or TKA occurs in 1.3% of patients, 26% of them being revised [3]. The economic burden of periprosthetic joint infection (PJI) is expected to exceed 50% of the inpatient resources spent in revisions by 2016 for TKA and by 2025 for THA [4]. This constitutes a substantial economic burden on patients, physicians, hospitals, health-care systems, and society as a whole.

The optimization of existing resources compels healthcare professionals to analyze in depth—and critique—the value of different therapeutic methods and technologies to provide cost-effective high-quality care. It is very important to correlate outcomes with the expenses incurred to achieve them. The identification and valuing of costs is often an additional step of the decision-making process when evaluating multiple competing strategies. Differential risks and balancing costs and benefits with various potential outcomes, especially when substantial uncertainty exists or when the timing of subsequent events is important, must be taken into account. Economic and decision analyses are evidence-based tools to guide healthcare choices. The guidelines these economic analyses provide have been reported in the literature and must be understood and used in order to adequately compare procedures and choose the best option [5, 6].

While the favorable cost effectiveness of a primary or revision THA/TKA has been demonstrated [7, 8], there is not the same certainty regarding the management of PJI [9, 10]. Determining the less expensive therapeutic methods that may best control infection and at the same time improve outcomes by minimizing patient morbidity and mortality might yield the highest quality of life for patients and the lowest economic impact on the healthcare systems as on society. This review summarizes the existing economic literature base, assesses the value of available data, and reports the less costly and more effective procedures for the treatment of PJI.

## 2. Prevention of Periprosthetic Infections

Prevention remains the least expensive approach against periprosthetic infection. The attainment of effective, low-cost, safe, and easy-to-use methods to elude periprosthetic infection is certainly the most logical methodology. 

The efficacy and cost effectiveness of antibiotics to prevent PJI depend on the antibiotic in case, the required quantity per dose, and the number of doses. With a similar efficacy, safety, and prices, a prophylactic regimen with cefazolin (1987 US$6.55/g)—a first-generation cephalosporin, one preoperative dose of 1 g followed by 500 mg every eight hours for six doses, saved cost when compared to a regimen with cefamandole (1987 US$6.99/g), a second-generation cephalosporin, 2 g preoperatively and then 1 g every eight hours for six doses (1987 US$26.20 versus US$55.92) [11].

The efficacy of a single-dose or short-term prophylaxis regimen has been estimated as equivalent to that of a long-term regimen, but with an associated reduction of risk of adverse effects and bacterial resistance and with lower costs [12]. In 1986, whether cefazolin was administered as a single 1 g parenteral dose intraoperatively or repeatedly every six hours for 24 hours, 48 hours, or seven days, the cost savings of an intraoperative antibiotic regimen versus a 48-hour regimen would have been US$77 per case. Changing from a seven-day regimen to a one-dose antibiotic, the savings would have been US$297 per patient without any difference in the infection rate [13]. The cost savings with current prices of these antibiotics could be US$31.45 per case of one dose versus a 48-hour regimen and US$110.04 per case using one dose instead of the seven-day regimen. 

There is no evidence to suggest that new-generation cephalosporins or the administrations of antibiotics beyond 24 hours postoperatively are more effective at preventing postoperative PJI in THA/TKA surgery than first-generation cephalosporins or single-dose or short-term administration. The use of one-dose first-generation cephalosporin is effective enough, reducing costs, risk of toxicity, and the development of bacterial resistance [12, 14].

The comparison between systemic administration of antibiotics and the use of antibiotic-loaded cement in order to prevent PJI has turned out inconclusive for a long time [12]. A favorable effect of adding antibiotics to the bone cement has been reported in the literature [15]. A cost effectiveness study [16], with a level II of evidence, has reported that antibiotic-impregnated bone cement in primary THA is cost-effective, avoiding revision due to infection, whereas the cost of revision is more than 3.5 times the cost of primary THA and in patients younger than 71 years, among other circumstances. The estimated cost of a 40 g packet of antibiotic-impregnated bone cement at the authors' institution was approximately 2002 US$365, while standard bone cement cost approximately 2002 US$65. Two packets of cement are used on average, resulting in an additional cost of 2002 US$600 per primary THA. The infection rate assumed in the model was 0.7% over ten years using standard bone cement and 0.4% with antibiotic-impregnated bone cement. A higher risk of infection from baseline makes the option of using antibiotic-impregnated bone cement even more cost-effective. A study carried out in 2011 determined the cost-savings of an initiative which aimed to reduce the costs of infection-related revisions by 10%. The savings were over US$70,000 [17]. 

## 3. Treatment of Periprosthetic Infections

Two high-level quality studies [19, 20] were analyzed. The treatment of patients with PJI is associated with significantly greater resource utilization compared with patients who underwent a primary or aseptic revision of TJR, which constitutes a substantial economic burden for patients, taxpayers, and hospitals. PJIs often require multiple reoperations, the prolonged use of antibiotics, a longer rehabilitation period, and frequent follow-up visits. Revision procedures for PJI are associated with a significant higher number of hospitalizations, hospital days, and number of operations, as well as a longer operative time, more blood loss, a lengthier antibiotic therapy, a higher number of radiographic examinations, and more total outpatient visits during the twelve-month period following the index procedure. A higher number of complications is also common. In general, in the case of an infected TKA, these parameters and costs were 3 to 4 times that of a primary TKA and more than twice that of an aseptic revision [21]. Sculco estimated an average cost of 1993 US$50,000 to $60,000 per case of infected THA [22]. 

The cost of hospital stay was the more relevant component of the whole set. In the Durham Regional Hospital (NC, USA), during the 90s, the total direct cost of hospitalization was estimated as an average of US$8206 for infected TJR versus US$5492 for uninfected arthroplasties [23]. In the Kraków Jagielloński University (Poland), in 2005, the direct cost of hospitalization for infected TJR reached US$37,903, and the cost of antibiotic treatment was US$11,067 [24]. In the Hospital of the University of Lund (Sweden), the cost of hospitalization was 1988 US$2530 for primary TKA versus US$33,663 for infected TKA; the cost of operation was US$3684 and US$10,411, respectively, and the cost of antibiotic therapy was US$65 and US$3778, respectively [25]. 

 There were also notable differences between the costs generated by the use of antibiotics during hospitalization and those of antibiotics administered to outpatients. In TKA infections, the average length of hospital treatment was 157 days against the 850 total days of outpatient treatment. The effort to reduce inpatient treatment, even if the outpatient treatment is long, has a notable impact on the total cost. In this sense, the trend of treatment of PJI would be directed towards the use of oral antibiotic therapy in outpatients, reducing hospital stays essentially to those related to surgical procedures.

## 4. Options in Surgical Techniques and Cost

Surgical options for treatment include debridement and retention of the prosthesis (DR), one- or two-stage exchange (OSE and TSE), resection arthroplasty, arthrodesis, and amputation. The success rates in eradication of PJI were below 50% with DR in retrospective series, but over 70% in the prospective modern studies with an optimal use of antibiotics [26, 27]. When prosthetic components are mechanically stable, symptoms have lasted three weeks or less, soft tissues are in good condition, and an agent active against the specific germs is available, an adequate implant DR achieving an 82%–100% cure rate of infection after three to six months of systemic therapy with ciprofloxacin and rifampin, as compared with a 58% cure rate with ciprofloxacin and placebo [27]. Control of acute PJI with DR and an adequate antibiotic regimen has been reported in 87%–89% of cases recently, but only when the bacteria is not multi resistant [28, 29].

Exchange arthroplasty is supported by many studies but has a higher rate of surgical morbidity and is more expensive than DR. In a systematic review of longitudinal studies with series of more than 50 patients, the success rate to eradicate PJI in THA was reported to be between 73.6% and 96.7% for OSE and between 87.7% and 95.1% for TSE, depending on the different authors. The random-effects analysis showed the rates of reinfection after one- and two-stage revisions were 10.56% and 8.71%, respectively [9]. In a recent meta-analysis, reinfection occurred with an estimated absolute risk of 13.1% with OSE and 10.4% with TSE [32].

In a decision-analysis, assuming that success of a given procedure was a period greater than 2 years without additional surgery, OSE might be the best solution for an acute THA infection and could lead to the greatest health-related quality of life, whereas the failure rate of DR is greater than 40% and the success rate of OSE is 66% or higher. With less than 38% success of DR and less than 69% success of OSE, TSE might lead to the greatest health-related quality of life [33].

The economic effects of OSE and TSE differ considerably. Although OSE may require a long hospital stay to administrate parenteral antibiotic therapy, the main determinant of cost is the requirement for additional surgery in TSE, with a cost 1.7 times more than OSE [30]. A short interval until reimplantation (two to four weeks) could allow both procedures to be performed during a single hospitalization [34].

The clinical and cost effectiveness of DR outcomes and TSE, with a median time to reimplantation of 2 months (range 1–12 months), in 65-year-old and frail 80-year-old patients with infected THA have been compared [33]. Patients who underwent initial DR were subjected to more additional operations than those who had initial exchange arthroplasty (3.2 versus 2.4 on average). In all cohorts, initial TSE provided a higher rate of infection-free survival than initial DR. However, the quality-adjusted life expectancy associated with DR was greater than with TSE only when old and frail population was considered. Incremental cost effectiveness ratio of DR compared with initial exchange arthroplasty was 1999 US$19,700 per QALY gained for 65-year-old men, US$21,800 per QALY gained for 65-year-old women, US$500 per QALY for 80-year-old men, and US$8200 per QALY for 80-year-old women. Initial DR became a cost-saving strategy relative to exchange arthroplasty when age at initial diagnosis of infection was over 80 years, when indirect and patient time costs were included in the analysis, and when the annual rate of infection recurrence after debridement was less than 19%. Even if the annual relapse rate after exchange arthroplasty was as low as 0.6%, initial DR remained cost-effective for patients over 80 years. The authors conclude that debridement and retention is a reasonable strategy for the treatment of PJI in patients over 80 years, staphylococcal or streptococcal infection, and well-fixed prosthesis.

For TKA, the efficacy of the different approaches to heal PJI is 20% for antibiotic therapy alone, 24% for debridement of soft tissue, 50% for resection arthroplasty, 76% for exchange arthroplasty, 90% for arthrodesis, and 100% for amputation [25]. In a systematic search of the literature about infected TKA, the overall success rate of PJI eradication was 73%–100% after OSE and 82%–100% after TSE, with 12–122 months follow up [35]. The clinical outcome (knee scores and range of motion) of OSE was no different from that of TSE.

From the perspective of hospitals which run their own operating rooms, the net financial impact range from cost savings is between US$5.09 and US$36.15 per case. In the case of surgeons who rent operating room space, the 24 minutes gained in operative time amount to a reduction in fees of approximately US$1794.91 per case, and when the cost of the device is included, net savings may be estimated in 2011 to be US$1294.91 per case [36].

The use of an antibiotic-loaded spacer in the TSE treatment of infected THA provides better infection control with good functional results and is superior a spacer-free two-stage treatment. The recurrence of infection was significantly higher without spacer (33.3% versus 10.5%). The use of a spacer increased the surgical time of the first stage by 40.1 minutes; but reduced the mean duration of the second surgical stage by 1 hour because reimplantation is easier; the surgical planes are found faster, the bone structures are well identified, and the bed for the prosthesis is accurately prepared. The stay in the intensive care unit after the second surgical stage was shorter when using a spacer (average, 1.4 days versus 4.1 days). Patients without a spacer stayed in hospital almost twice as long as patients with a spacer because a period of skeletal traction is mandatory to allow healing of the soft tissues maintaining the length of extremity as much as possible.

Another way to realize cost savings in the treatment of PJI is to use the liquid form of gentamicin mixed with the bone cement fixing the prosthetic components or filling the cement spacers employed in TSE. It is the most widely and readily available antibiotic for mixing bone cement, and much less costly (US$4 for a 480 mg dose) than tobramycin (US$120–310 per 1.2 g dose) and than the powdered form of gentamicin, which is at least as expensive as tobramycin [37, 38]. The limitation imposed by using liquid gentamicin in the bone cement fixing prosthesis is a decrease of mechanical properties of the cement produced, but this is irrelevant for the temporary cement spacers. If tobramycin is replaced by liquid gentamicin in bone cement spacers, an annual antibiotic cost saving of US$7,400,000 could be achieved in the United States alone [38].

From the current literature, a certain consensus emerges regarding complete cost coverage of PJI treatment not being feasible in most healthcare systems. An estimated average loss is of approximately $15,000 per case for the total group of patients as a whole treated for infected TKA, between $30,000 per case per Medicare patient in USA in 1993 [21], and 7745 per case in Germany [39]. Furthermore, inflation decreased the value of estimated mean reimbursement per hospitalization for PJI in USA from 2004 US$9746 in 1997 to US$8719 in 2004 [40]. The lack of incremental reimbursement for these procedures discourages physicians and hospitals from treating patients with PJI [41] (Table 1). Reimbursement to both hospitals and physicians should be more accurate and reflect the actual magnitude of resources consumed by these patients. 

 Determining the treatment that best controls infection minimizing patient morbidity and mortality with the less cost possible may offer the best solution to the problem. Among the protocols and techniques currently used to reduce the incidence and to treat PJI, there is not a clear option. The resource allocation and financial costs of treating PJI in orthopedic surgery can often rise 3–13 times more than the cost of the index procedure, thus making PJI an ideal target for cost-effective solutions in a value-driven healthcare model [18]. The recommendations set out by different authors [34, 42] and proven useful when choosing the method of treatment of PJI [43] must be tempered in view of results of these economic studies (Table 2).

## 5. Level of Evidence

There are only a few high-quality studies dealing with the accurate evaluation of cost effectiveness of PJI treatment. The lack of level-I evidence studies regarding interventions in PJI has made it difficult to perform high-quality cost-utility analyses. The difficulties and ethical concerns of performing randomized studies in this field are evident, more so as they are related with treatment procedures. The number of patients needed to carry on correctly such studies is yet another concern. It has been estimated that 7,000–14,000 patients would be needed to demonstrate a 20% reduction in infection rate if the baseline infection rate was 5% [44]. A powered study assuming a 1.5% to 2.0% infection rate would require in the range of 10,000 patients to determine the effect of any one independent variable, with power greater than 80% [45]. In order to show a 50% reduction in an infection rate of 2%, for example, at the 5% significance level and 80% power, over 2,300 patients would be required in each treatment arm [12]. Multiple variables would require at least 70,000 patients [45]. Given the low PJI rates, it may not be cost-effective to carry out mega-trials in this area. It is necessary to analyze risk factors to identify high-risk groups on whom profitable high-quality studies of new or additional prophylactic, diagnosis, or treatment measures could be performed with sufficient power to achieve a statistically significant difference.

Most studies in this field have estimated charges or costs of management of PJI assuming partially the actual price of actions. The direct medical costs, length of hospitalization, and total hospital costs were the most frequently considered parameters as indicators to evaluate resource utilization [21, 39, 46], while outpatient charges, the costs associated with retreatment of a failed treatment, and the indirect costs associated with lost wages and productivity only sometimes were accounted for [19, 25, 46, 47]. The use of the direct costs of hospitalization has been suggested as the best method to estimate the costs related to infection treatment, since they represent the real costs to the hospital for the items and services used by each patient, but this approach probably underestimates the total resource utilization and also misjudges the overall financial and personal impact of PJI on the patients themselves [48].

On the other hand, charges are only a proxy for cost and an inaccurate measure of health-care resource utilization for many reasons, essentially due to the fact that the economic basis of charges differs substantially among health-care facilities and geographic locations. Impact on functional outcomes, working and daily activities, quality of life, and well-being should also be considered. Thus, the burden on patients of PJI could far exceed the costs usually evaluated in this kind of studies.

## Figures and Tables

**Table 1:** Cost of noninfected TJR and debridement and retention for treatment of infected TJR [18].

	Non-infected TJR	Infected TJR DR	*P *
Total inpatient	22,688	57,494	0.001
Medical	1732	9117	0.001
Nursing	7830	28,140	0.001
Operating room	11,173	18,977	0.001
Implants	7468	8336	0.3
Intensive care unit	0	0	1.0
Allied health	1562	3707	0.001
Medical imaging	64	278	0.001
Pathology	188	1710	0.001
Pharmacy	331	2388	0.001
Hospital at home	469	1624	0.02
Total outpatient	377	4426	0.001
Medical	23	901	0.001
Nursing	278	442	0.03
Allied health	0	44	0.002
Medical imaging	0	120	0.001
Pathology	0	146	0.001
Pharmacy	0	1846	0.001
Total emergency	0	553	0.001
Total costs	24,073	75,661	0.001

**Table 2:** Different options of treatment of PJI. Estimated average cost in 2012 adjusted currencies and normalized to US$.

Author (date)	Debridement and retention	One-stage revision	Two-stage revision	Resection arthroplasty	Arthrodesis	Amputation
TJR (THA + TKA)						

Peel et al. (2013) [18]*	75,661					

THA						

Fisman et al. (2001) [19]*	74,015		70,634			
Klouche et al. (2010) [30]*		43,586	75,737 × 1.7			

TKA						

Hebert et al. (1996) [21]**			150,984	121,866	101,346	347,789
Lavernia et al. (2006) [31]**		133,970	134,670	113,575		

*Total hospital costs.

**Total hospital costs + total outpatient costs.
